# A systematic review of modelling approaches in economic evaluations of health interventions for drug and alcohol problems

**DOI:** 10.1186/s12913-016-1368-8

**Published:** 2016-04-13

**Authors:** Van Phuong Hoang, Marian Shanahan, Nagesh Shukla, Pascal Perez, Michael Farrell, Alison Ritter

**Affiliations:** Drug Policy Modelling Program, National Drug and Alcohol Research Centre, University of New South Wales, Sydney, NSW 2031 Australia; SMART Infrastructure Facility, University of Wollongong, Wollongong, NSW 2522 Australia; National Drug and Alcohol Research Centre, University of New South Wales, Sydney, NSW 2031 Australia

**Keywords:** Substance abuse, Drug dependence, Alcohol dependence, Economic evaluation, Modelling

## Abstract

**Background:**

The overarching goal of health policies is to maximize health and societal benefits. Economic evaluations can play a vital role in assessing whether or not such benefits occur. This paper reviews the application of modelling techniques in economic evaluations of drug and alcohol interventions with regard to (i) modelling paradigms themselves; (ii) perspectives of costs and benefits and (iii) time frame.

**Methods:**

Papers that use modelling approaches for economic evaluations of drug and alcohol interventions were identified by carrying out searches of major databases.

**Results:**

Thirty eight papers met the inclusion criteria. Overall, the cohort Markov models remain the most popular approach, followed by decision trees, Individual based model and System dynamics model (SD). Most of the papers adopted a long term time frame to reflect the long term costs and benefits of health interventions. However, it was fairly common among the reviewed papers to adopt a narrow perspective that only takes into account costs and benefits borne by the health care sector.

**Conclusions:**

This review paper informs policy makers about the availability of modelling techniques that can be used to enhance the quality of economic evaluations for drug and alcohol treatment interventions.

**Electronic supplementary material:**

The online version of this article (doi:10.1186/s12913-016-1368-8) contains supplementary material, which is available to authorized users.

## Background

### Economic evaluations and the role of modelling

The economic, social and health consequences related to the consumption of drug and alcohol are a global concern with 0.8 % of the global all-cause disability adjusted life years (DALYs) attributable to illicit drugs and alcohol [1]. Governments are faced with difficult decisions of how to best to allocate the limited available resources in order to maximise the value of spending (e.g. to achieve the highest net/social benefit). Modelling is frequently being applied as a method of depicting the complexity of decision making with respect to interventions and to more accurately capture associated costs and benefits in economic evaluations.

Modelling can be broadly defined as the reproduction of events and possible consequences due to alternative policy options at the cohort or individual levels using mathematical and statistical framework. In the drug and alcohol area, models have been developed, for example, to mimic the heroin use and treatment profile of individuals over their life time, from which realistic outcomes can be calculated [2, 3]. Despite modelling being widely applied for analysis of treatment for alcohol and drug problems, comprehension about which models are suitable to use for which research questions and how to interpret the results and caveats of the models remains limited. The primary aim of this paper, therefore, is to review the use of economic modelling in the evaluation of drug and alcohol treatment. Three modelling aspects are the focus of this review: (i) modelling approaches, (ii) perspectives of costs and benefits, and (iii) time frame (e.g. short or long-term time frame). Additionally, the concerns and relevance of each type of model will be discussed.

Decisions about the perspective of costs and benefits may have substantial impacts on the modelled outcomes due to the multiplicity of effects of interventions such as crime, transmission of infectious diseases, and other long-term consequences of drug and alcohol dependence. We adopt four distinct perspectives of costs and benefits according to common definitions in health economics as follows: *“(i) individual patient — all consequences that accrue to the patient and all costs that are borne by the patient; (ii) health funder — for example, the costs that fall on a State health authority; (iii) health care sector — all costs that fall to the health sector, including, but not limited to, hospitals, specialists, general practitioners, ancillary services and community services (includes all health improvements or health-related quality-of-life improvements but does not include such things as informal carer costs, patient transport costs or time off work); and (iv) society — all costs and consequences that arise from the options no matter who pays or who receives benefits from them”* [4]. In addition, different types of interventions may generate immediate or long-term effects. Therefore, for an economic evaluation to provide relevant policy advice, it should carefully account for the major costs and benefits and relevant time-frame associated with each intervention.

### Modelling classifications

Economic evaluation is defined as a tool to compare the costs and benefits of alternative interventions, treatments or policy options. There is a suite of evaluation methods in health care research that may be used for different purposes. The search for information on efficiency often leads to an economic evaluation being conducted. Typically these have been cost-effectiveness (CEA) studies [5–7] or cost-utility (CUA) studies [8, 9]. While CEA and CUA studies can provide evidence to make the case for the investment in one treatment over another, the typically short-term time frame of the studies in the drug and alcohol field (e.g. 3 to 12 months) [10, 11] and the necessity to choose a single outcome means that they can fail to capture the full spectrum of costs and benefits. The single outcome, the narrow perspective taken by many studies and the exclusion of externalities such as costs of crime has led some to call for a greater focus on cost benefit analyses (CBA) when assessing interventions in the drug and alcohol field [12].

Cost-benefit analysis (CBA), with their outcomes valued in monetary terms, allows for the inclusion of a broader range of outcomes and externalities. It is, however, this inclusion in the CBA of a range of outcomes, and the necessity to value them in monetary terms that provides a number of challenges when conducting a CBA [13].

For those who are dependent on drug and alcohol there maybe movement in and out of treatment and prison, and the presence of co-morbid medical conditions such as anxiety disorders, depression, HIV/AIDs, Hepatitis C, and overdose [14–16]. Short-term economic evaluations conducted alongside RCTs are not usually sufficient to capture this multiplicity of outcomes. This problem has been long recognised when conducting economic evaluations of interventions for chronic illnesses. For example, in areas such as diabetes and heart disease there has been a shift to the use of economic modelling which can account for a multiplicity of outcomes and the long term and complex nature of the disease [17, 18].

The improvements in computer technology and development of new methods have made modelling a powerful tool to inform health policies. Through the synthesis of evidence from multiple datasets on the impact of interventions, behaviour changes of the individual and externalities, models can be constructed to compare the alternative interventions over a long timeframe. In general, modelling offers several advantages including: (i) extrapolating beyond the data observed in a trial; (ii) linking intermediate clinical endpoints to final outcomes; (iii) generalizing to other settings; (iv) synthesizing head-to-head comparisons where relevant; and (v) informing decisions in the absence of hard data [19]. Broadly, a model includes four components: (i) the individuals/cohort under study; (ii) the states; (iii) the rules of transition between states over time; and (iii) the associated costs and benefits attached in each state. The complexity of a model depends on how the model components are defined. There exist several classification systems for health economic models. For example, Brennan et al. [20] proposes a classification based on whether a model is cohort or individual-based, discrete or continuous time-dependent and whether or not it allows for interactions between individuals. As with many classification systems, simplifications are often necessary to nudge the models into fixed categories, and many models may actually cross boundaries. Table 1 provides examples of common modelling paradigms classified according to two criteria proposed by Brennan et al. [20].

**Table 1:** Classification of modelling paradigms

	Cohort-based	Individual-based
No interaction allowed	Decision tree Cohort Markov model	Individual-based microsimulation/Markov microsimulation
Interaction allowed	System dynamics model	Agent based model Discrete event simulation

Adapted from Brennan et al. [21]

Decision tree models are the simplest and historically the most widely used form of models in health economics: all patient pathways are clearly laid out with their associated probabilities and outcomes (i.e. costs related to events, mortality rate, and the probability of committing crime) which are entered at the terminal node. The mean value of each decision in the tree is calculated by summing probabilities associated with each outcome. These models are usually used when the time frame is short, the process is not complicated, reoccurring events are not important, there is little heterogeneity between individuals in terms of outcomes and there is no interaction between individuals [20–22].

Cohort Markov models are suited to circumstances where the order of events is important, events may repeat, and the events may occur over a longer period of time. However, in addition to not allowing interaction between individuals in the model, cohort Markov models do not allow the probability of transitioning to another state in the model to depend upon the time spent in that state or previous history [20]. System dynamic models are also cohort-based models but allow interaction among entities by modelling the rate of change of the system to be dependent on the system itself (i.e. feedback loops).

Individual-based models can simulate a richer heterogeneity of individual trajectories, allowing for more realistic and complex patterns of disease evolution to emerge. For example, Individual-based microsimulation/Markov microsimulation can simulate the life-time trajectories of participants and record participants’ history. The transitions across different states may be conditional on previous events/history that participants have gone through. Agent-based models (ABM) include autonomous agents whose behaviour depends upon their past and current internal states, as well as states from other agents and their environment. Discrete event simulations (DES) allow for both modelling individual trajectories and interaction between entities in the system [20].

It is apparent, even from these brief descriptions, that different models are appropriate for different purposes. For example, models which are being used to generate evidence surrounding communicable disease will need to account for interaction between agents (i.e. ABM, DES) whereas a brief intervention for low risk alcohol consumption may only require a decision tree model. In order for the results to be meaningful, the choice of a modelling paradigm needs to reflect the problem being addressed and its context.

## Methods

Papers that use modelling approaches for economic evaluations of drug and alcohol health interventions were identified by carrying out searches on major databases including: SCOPUS, Medline, CINAHL, PyscInfo, EconLit and ISI Web of Sciences. The search terms were: (drug dependence treatment OR substance abuse OR heroin OR methadone OR buprenorphine OR cocaine OR Methamphetamine OR Cannabis OR opioid dependence OR alcohol OR dependence) AND (economic evaluation OR cost-effectiveness OR cost-utility OR cost-benefit) AND (model* OR simulation). The inclusion criteria were: (i) economic evaluation studies of drug and alcohol health interventions; (ii) incorporating a modelling approach; (iii) peer-reviewed articles in academic journals published in the period from database inception to September 2015 in the English language. The exclusion criteria, therefore, were papers that were not economic evaluation, did not use modelling or were not about drug and alcohol health interventions. Accordingly, there were drug and alcohol modelling studies that were not met inclusion criteria such as: the simulation of the life course of cannabis use [23]; the simulation of Australian drug markets [24]; the examination of the impact of ecstasy pill-testing on the prevalence of harms [25] and exploring policy options and risks for alcohol consumption [26, 27].

Ethics for this study was not required because this study involved the analysis of published literature.

**Fig. 1:**
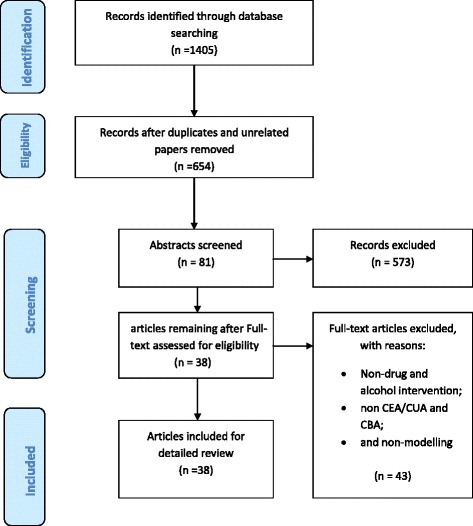
Flow chart of literature search

The initial search of databases yielded 1405 papers; 751 duplicate and unrelated papers were eliminated by checking titles, resulting in 654 papers. Next, each abstract was reviewed during which 81 papers were selected. The final step was to conduct in-depth content review of the selected papers with respect to modelling approach and economic evaluation framework. Those papers that were not a cost-benefit, a cost-effectiveness, or a cost-utility analysis of a health intervention for drug or alcohol problems, and did not use modelling methods were excluded (*n* = 43 exclusions). Finally, 38 papers were included in the review (see Fig. 1 and Additional file 1). During all screening steps, a set of papers was allocated to two authors so that each paper was screened independently twice. Any discrepancy in the selection of papers was discussed between the authors to reach a mutual agreement.

**Table 2:** Classifications of reviewed papers

Models used		Decision Tree (*n* = 11)	Cohort Markov (*n* = 20)	Individual-based microsimulation Models (*n* = 2)	System Dynamics Model (*n* = 3)	Other Models (*n* = 2)	Total (*n* = 38)
Design		- Aggregate	- Aggregate	- Individual	- Aggregate	- Aggregate	
		- No accounting for heterogeneity	- Limited heterogeneity by using states	- Flexibility to include heterogeneity by using transition probability function	- Limited heterogeneity by using states	- No heterogeneity	
		- Untimed	- Timed	- Timed	- Timed	- Untimed: only before and after intervention	
		- No history	- No history	- History	- No history - Allow interactions	- No history	
Perspective of Costs and Benefits	Health care sector	6	17	0	3	0	26
	Societal	5	3	2	0	2	12
Timeframe	0-1 year	5	1	0	0	0	6
	1-10 years	3	5	0	1	0	9
	10 years to life time	3	14	2	2	2	23

**Table 3:** Summary of papers and classification of modelling approaches, timeframe and perspectives in costs and benefits

Authors/year of study and summary	Country	Year of study	Analytic method	Study participants	Modelling approach	Time frame	Perspective on costs and benefits
Barbosa et al. (2010) [41] have used a cohort based probabilistic lifetime Markov model where alcohol consumption and drinking history are used for classifying patients into 4 Markov states. One year cycle length was used for the model. The main outcomes were QALYs and lifetime costs.	The U.K	2010	CEA	Males who are seeking alcohol treatment	Markov	Life time	Health care sector
Barnett et al. (2001) [8] have developed a dynamic compartmental model to estimate the effect of adding buprenorphine maintenance therapy to the US healthcare system. The model divides population into mutually exclusive groups (“compartments”) based on HIV status and drug use status. Transitions between these compartments were modelled as a system of non-linear differential equations. Current healthcare costs and outcomes with the adoption of buprenorphine under different scenarios were compared.	The U.S	2001	CEA	Current population of methadone treatment participants in the U.S health care system	Markov	10 years	Health care sector
Coffin and Sullivan (2013) [42] employs integrated cohort based Markov and decision analytic model to assess cost-effectiveness of distributing naloxone to heroin users in the U.S over lifetime. In the model, heroin users enter the model in ‘Heroin use’ state and can make transitions to ‘discontinue and relapse’, ‘overdose’, or ‘death by other reasons’ state. The ‘overdose’ state triggers a decision tree based model based on naloxone distribution to assess whether an individual will survive or die after overdose.	The U.S	2013	CEA	Hypothetical 21-year-old novice U.S. heroin user	Markov	Life time	Societal
Downs and Klein (1995) [28] developed cost-effectiveness model based on decision trees for adolescent population (15-19 years). The intervention is screening visits for alcohol abuse and unsafe sexual activity.	The U.S	1995	CEA	Adolescents aged 15 to 19 years	Decision trees	5 years	Societal
Magnus et al. (2012) [39] modelled economic and health gains on the basis of an absolute change in alcohol consumption. They modelled population simulation model to determine lifetime benefits of a reduction in per capita alcohol consumption from 2008 Australian adult cohort (aged ≥ 15 years). It considers workforce production gains model, household production and leisure time model, and health sector cost estimates for economic benefit evaluation. This study aims to evaluate the benefits of reduction of alcohol use thanks to a hypothetical intervention.	Australia	2012	N/A	The 2008 Australian population	Aggregate model	Lifetime	Societal
Navarro et al. (2011) [29] developed a decision tree based model to assess outcomes and costs of GP-delivered intervention for alcohol misuse. Nine difference scenarios with incremental increase in screening, brief intervention, or in the combination of screening and brief intervention were compared to current practice.	Australia	2011	CEA	Risky drinkers in 10 rural communities in New South Wales, Australia	Decision trees	1 year	Health care sector
Purshouse et al. (2013) [30] developed a health economic model combining the healthcare resource requirements for alcohol screening and brief intervention with an epidemiological model of relationships between alcohol consumption and health harms.	England	2013	CEA	Risky drinkers who are screened through GP’s visits	Decision trees	30 years	Health care sector
Sheerin et al. (2004) [40] used Markov model to model cohorts of injecting drug users, changes in their health states and effects of methadone maintenance therapy and anti-viral therapy on morbidity and mortality.	New Zealand	2004	CEA	Injecting drug users (IDUs)	Markov	Lifetime	Health care sector
Wammes et al. (2012) [59] used Asian epidemic model and resource needs model to evaluate the long term preventive impact of expanding methadone maintenance therapy in West Java. In this model, population is divided into 8 compartments and individuals move from one compartment to another based on a transition probabilities.	Indonesia	2012	CEA	Injecting drug users (IDUs)	Markov	20 years	Societal
Tran et al. (2012) [32] developed a simulated decision tree based model to represent HIV-positive drug user’s transition to 4 health state within one year horizon. Each of the states had services cost and health outcomes which were used for cost and benefit assessments.	Vietnam	2012	CEA	HIV-positive drug users	Decision trees	1 year	Health care sector
Zaric et al. (2000) [43] developed a dynamic compartmental model to assess the effects of increased methadone maintenance capacity on healthcare costs and survival (QALYs) for HIV epidemic in a population aged 18-44years. Population is divided into 9 subgroups based on risk group and HIV infection status. Size of each compartment over time is modelled with the help of set of non-linear differential equations.	The U.S	2000	CEA	The population of adults, aged 18 to 44	Markov	10 years	Health care sector
Tariq et al. (2009) [44] used a RIVM model to conduct a CEA of screening and brief intervention for alcohol in primary care targeting at reisk drinkers; outcomes were ICER, costs and QALY.	The Netherlands	2009	CEA	Risky drinkers aged between 20 and 65 who visit the GP yearly (50 %)	Markov	80 years	Health care sector
van den Berg et al. (2008) [45] used chronic disease model (CDM) to estimate the cost effectiveness of an alcohol tax increase from a health care perspective in the Netherlands; the outcomes were QALYs and LYS and health care costs	The Netherlands	2008	CEA	Current Dutch population	Markov	100 years	Health care sector
Vickerman et al. (2012) [36] used a system of differential equations to examine the impact on Hepatitis C of scaling up OST and needle syringe programs;	The U.K	2012	CEA	Injecting Drug Users (IDUs)	System dynamics	20 years	Health care sector
Nosyk et al. (2012) [46] used a semi Markov cohort model to assess the increemental cost effectiveness of methadone versus diacetylmorphine in a cohort who had multiple failures of OST ; used data from the North American Opiate Medication Initiative trial; Outcomes used were QALYs and social costs (treatment, HIV, crime, calculated an ICER)	Canada	2012	CEA	Injective drug users (IDUs)	Markov	Life time	Societal
Zaric and Brandeau (2001) [37] used an epidemic model to determine optimal allocation of HIV prevention funds. Three types of programs NSP (1), methadone (2), and condoms (3). Outcomes were QALYs gained; and the investment portfolio that maximises the number of HIV cases averted	The U.S	2001	Resource allocation framework	a population of injection drug users (IDUs) and non-IDUs	System dynamics	3 years	Health care sector
Mortimer and Segal (2005) [47] used a time dependent state-transition model to compare complementary and competing interventions for prevention or treatment of alcohol misuse and dependence; compares usual care with interventions. Assesses proportions of patients drinking beyond specified threshold, at 6,12 months follow-up; costs; cost utility; used QALY league tables	Australia	2005	CEA	Problem alcohol drinkers	Markov	Life time	Health care sector
Palmer et al. (2000) [48] uses a Markov model to explore the long term clinical and economic outcomes of alcohol maintenance with counselling or counselling plus accamprosate. Discounted and non-discounted LE and life time costs, incremental cost effectiveness; uses abstinence.	Germany	2000	CEA	Problem alcohol drinkers	Markov	Life time	Health care sector
Zaric et al. (2000) [49] uses a dynamic compartmental model of HIV to assess the cost effectiveness of MMT as a method of preventing HIV infection; the outcomes of the model are discounted LYS and QALYs and discounted health care and treatment costs	The U.S	2000	CEA	Injective drug users (IDUs)	Markov	10 years	Health care sector
Adi et al. (2007) [31] investigates the clinical effectiveness and cost effectiveness of naltrexone for relapse prevention in detoxified opioid dependent persons compared to psychosocial support.	The U.K	2007	CEA	Injective drug users (IDUs)	Decision trees	1 year	Societal
Barnett (1999) [50] examined cost effectiveness of methadone compared to standard care among cohort of 25 years old heroin users in the U.S.	The U.S	1999	CEA	Injective drug users (IDUs)	Markov	Life time	Health care sector
Bayoumi (2008) [51] examined cost effectiveness of medically supervised injecting centre; compared situation with supervised injecting centre to no injecting centre but with needle syringe programs.	Canada	2008	CEA	Injection drug users and persons infected with HIV and hepatitis C virus	Markov	10 years	Health care sector
Alistar et al. (2011) [60] have developed a dynamic compartment model of a population of IDUs on methadone substitution therapy, IDUs injecting opiates and non-IDUs in order to evaluate the effectiveness and cost effectiveness of expanding methadone substitution therapy to IDUs, increasing access to ART, or both. The outcome measures are the cost-effectiveness and QALYs.	Ukraine	2011	CEA	A population of non-IDUs, IDUs who inject opiates, and IDUs in MMT, adding an oral PrEP program (tenofovir/emtricitabine, 49 % susceptibility reduction) for uninfected IDUs	Markov	20 years	Health care sector
Kapoor et al. (2009) [52] examine cost-effectiveness of various screening strategies for unhealthy alcohol use with % Carbohydrate Deficient Transferrin using a Markov model.	The U.S	2009	CEA	Adult men and women (ages 18 to 100 years) in primary care	Markov	Life time	Health care sector
Schackman et al. (2015) [62] evaluate the cost-effectiveness of long-term office-based buprenorphine/naloxone treatment for clinically stable opioid-dependent patients compared to no treatment.	The U.S	2012	CEA	Cohort of clinically stable opioid-dependent individuals who have already completed 6 months of office-based buprenorphine/naloxone treatment	Markov	2 year	Health care sector
Tran et al (2012) [33] analyse the cost-effectiveness and budget impact of the methadone maintenance treatment (MMT) programme in HIV prevention and treatment among injection drug users (DUs) in Vietnam.	Vietnam	2012	CEA	injection drug users (DUs)	Decision trees	1 year	Health-care sector
Zarkin (2012) [3] builds a Discrete event simulation to estimate the net societal benefits of diverting eligible poisoners to community based treatment in the U.S.	The U.S	2012	CBA	A cohort of individuals who are incarcerated in the state prison system in the United States	Discrete event simulation	Life time	Societal
Zarkin et al. (2005) [2] estimate net societal benefits of providing methadone treatment in the U.S using Monte Carlo simulation model.	The U.S	2005	CBA	The general population aged 18–60 (a percentage is heroin users)	Individual-based microsimulation	Life time	Societal
Rydell et al. (1994) [61] presents a model that estimates the relative cost-effectiveness of four cocaine-control programs: three "supply control" programs (source-country control, interdiction, and domestic enforcement) and a "demand control" program (treating heavy users).	The U.S	1996	CEA	The market includes the supply and demand of cocaine	Aggregate model	15 years	Societal
Cartwright (2000) [34] estimates the benefits of reduced cocaine consumption in terms of reduced societal costs resulting from the introduction of a medication for cocaine dependence with a small incremental treatment effect.	The U.S	2000	CBA	Heavy cocaine users	Decision trees	1 year	Societal
Ciketic et al. (2015) [53] evaluates the cost-effectiveness of counselling as a treatment option for illicit MA use compared with no treatment option.	Australia	2015	CEA	Individuals recruited into Methamphetamine Treatment Evaluation Study (MATES)	Decision trees	3 years	Societal
Alistar et al. (2014) [54] estimated the effectiveness and cost effectiveness of strategies for using oral PrEP in various combinations with methadone maintenance treatment (MMT) and antiretroviral treatment (ART) in Ukraine, a representative case for mixed HIV epidemics.	Ukraine	2014	CEA	A population of non-IDUs, IDUs who inject opiates, and IDUs in MMT, adding an oral PrEP program (tenofovir/emtricitabine, 49 % susceptibility reduction) for uninfected IDUs.	Markov	20 years	Health care sector
Angus et al. (2014) [55] adapt the Sheffield Alcohol Policy Model to evaluate a programme of screening and brief interventions (SBI) in Italy. Results are reported as Incremental Cost-Effectiveness Ratios (ICERs) of SBI programmes versus a ‘do-nothing’ scenario.	Italy	2014	CEA	General population who visit GPs	Decision trees	30 years	Societal
Jackson et al. (2015) [56] estimate the cost-effectiveness of injectable extended release naltrexone (XR-NTX) compared to methadone maintenance and buprenorphine maintenance treatment (MMT and BMT respectively) for adult males enrolled in treatment for opioid dependence in the United States from the perspective of state-level addiction treatment payers.	The U.S	2015	CEA	Adult males enrolled in treatment for opioid dependence	Markov	6 months	Health care sector
Laramee et al (2014) [57] investigate whether nalmefene combined with psychosocial support is cost-effective compared with psychosocial support alone for reducing alcohol consumption in alcohol-dependent patients with high/very high drinking risk levels (DRLs) as defined by the WHO, and to evaluate the public health benefit of reducing harmful alcohol-attributable diseases, injuries and deaths.	The U.K (England and Wales)	2014	CEA	The licensed population for nalmefene	Markov	5 years	Health care sector
Schackman et al (2015) [62] evaluate the cost-effectiveness of rapid hepatitis C virus (HCV) and simultaneous HCV/HIV antibody testing in substance abuse treatment programs.	The U.S	2014	CEA	Opioid users in substance abuse treatment programs	Decision trees	Life time	Health care sector
Thanh et al (2014) [63] used a decision analytic modeling technique to estimate the incremental cost–effectiveness ratio and the net monetary benefit of the Parent–Child Assistance Program (P-CAP) within the Alberta Fetal Alcohol Spectrum Disorder Service Networks in Canada.	Canada	2015	CEA	Women who abuse substances (e.g. alcohol and/or drugs) and are pregnant	Decision trees	3 years	Health care sector
Braithwaite et al (2014) [58] estimate the portion of HIV infections attributable to unhealthy alcohol use and to evaluate the impact of hypothetical interventions directed at unhealthy alcohol use on HIV infections and deaths.	Kenya	2014	CEA	The Kenyan population	System dynamics	20 years	Health care sector

## Results

Tables 2 and 3 present a summary of classifications of reviewed papers in regarding to: modelling approaches, perspectives on costs and benefits and time frame.

### Modelling approaches

In terms of types of model, decision trees were used in 11 papers (29 %), cohort-based Markov modelling in 20 papers (52 %), system dynamics in 3 papers (8 %), individual based model in 2 papers (5 %) and another model type in 2 papers (5 %). The majority of studies were conducted in the US (*n* = 15), the U.K (*n* = 5), Australia (*n* = 4), Canada (*n* = 3) and the remainder were from a range of countries. Almost all of the studies used cost-effectiveness analysis (CEA) as the analytical method (*n* = 32), two studies used cost-benefit analysis (CBA) and the rest used other analytical methods.

#### Decision trees

Eleven papers were found to have used a decision tree modelling approach. The topics ranged from short-term interventions for alcohol problem such as screening for alcohol users [28–30]; to relapse prevention for opioid use [31]; cost-effectiveness of methadone maintenance treatment (MMT) [32, 33] and providing medications to reduce cocaine dependence [34]. One paper, Navarro et al. [29] developed a decision tree model to assess outcomes and costs of a GP-delivered intervention for alcohol misuse for different scenarios (increase in screening; brief intervention; or a combination of screening and brief intervention and current practice). This paper, while providing an assessment of cost-effectiveness of different alternative interventions at an aggregate level, it does not take into account the role of individual attributes such as age, use and treatment history among the participants to determine the outcomes: effectively it assumes that the effect is the same for all participants. In the area of Opioid Substitution Treatment (OST), Tran et al. [32] developed a simulated decision tree model to evaluate cost-effectiveness of providing methadone to HIV-positive drug users in three treatment options; patients: (i) did not receive any drug substitution therapy during antiretroviral therapy (ART); (ii) received ART and MMT in standalone clinics, as currently delivered in Vietnam; and (iii) patients received ART under the direct supervision of health workers together with methadone during daily clinic visits. This model evaluated the outcomes at one-year after entering treatment and assumed the same treatment effects for all participants regardless of treatment history or use history. While these assumptions simplify the analysis and point to the key results, again they demonstrate the limitations in capturing the long term value of MMT treatment as well as the differential treatment effects across participants.

#### Cohort Markov Models

The majority of the reviewed papers used the cohort Markov modelling approach (*n* = 20) and cover a diverse range of drug and alcohol topics. One example of the application of cohort Markov model was an evaluation of cost-effectiveness of long-term outpatient buprenorphine-naloxone treatment for opioid dependence in primary care [35]. The cohort was allocated across four health states including ‘In Treatment Off Drugs’, ‘In Treatment On Drugs’, ‘Out of Treatment Off Drugs’, and ‘Out of Treatment On Drugs’. The participants were then transitioned across states based on fixed transition probabilities. The outcomes including costs of treatment and QALYs (a utility based quality adjusted life year measurement which takes the value of 1 if full health and 0 if death) were attached to the states over the course of 24 months. This model demonstrates the key evolutions of opioid dependence and treatment over time and sets a clear structure for economic evaluation of treatment but has several limitations that are common in cohort Markov models. The lack of personal attributes, opioid use history and treatment history when determining either transition probabilities or costs and benefits of treatment mean that the model may not reflect the heterogeneity of the heroin treatment population.

#### System Dynamics Model

System dynamics modelling is used to account for the interaction between individuals in the cohort. These models are commonly used for infectious diseases such as HIV/AIDs and hepatitis C. For example, system dynamics models have been used in assessing the role methadone treatment has in reducing risky behaviours and new infections, which further reduces the rate of HIV infection in the cohort (i.e. feedback loops). We found three papers that applied this approach. The first paper examines the effect of needle-syringe programmes and opiate substitution treatment on the reductions in hepatitis C virus prevalence in the UK [36]. The rates of infection are modelled through a system of differential equations that depend on susceptible and chronic injecting drug users in various interventions. The second paper evaluates the optimal investment to maximize life years gained or HIV infections averted in a population of infected drug users and non-users [37]. The optimization problem was set up with the objective of maximizing life years gained or HIV infections averted taking into account the non-linear growth of HIV infections. Despite the complexity of such models (and their relevance for infectious disease) one of the limitations of the system dynamics model is that it ignores individual attributes and history thus effectively limiting the heterogeneity of the cohort. However, the system dynamics modelling has an ability to depict the interaction among entities, which is crucial to obtain valid outcomes in the treatment and prevention of infectious diseases.

#### Individual Based Models

We found only two papers that met the review inclusion criteria for individual based microsimulation model. The first estimated the costs and benefits of providing methadone treatments in the context of the U.S. Here, Zarkin et al. [2] built an individual-based microsimulation that simulated the life time trajectories of a cohort of heroin users beginning at age 18 and ending at age 60. The participants cycled in and out of five mutually exclusive states including: (i) not using drugs and not in treatment; (ii) using drugs and not in treatment; (iii) in treatment; (iv) in prison and not using drugs and (v) in prison and using drugs. The key individual attributes such as age, gender, current heroin use, heroin use history and treatment history were used to determine the transition probabilities. While providing valuable results, the lack of data necessitated several simplifying assumptions. These included the assumption that the only treatment available was opioid substitution treatment and the transition probabilities were assumed to depend on only a few key individual attributes and heroin use and treatment history [2].

A second paper by Zarkin et al. [3] used a Discrete event simulation to assess the lifetime costs and benefits of diverting substance-abusing offenders from state prison to community-based treatment based on the premise that those offenders who undertook treatment have decreased criminal recidivism rates translating to costs-savings to the criminal justice system and gains from increasing probability of re-entering the labour market of offenders. This model captured the key events in the offenders’ lifetime such as substance abuse, criminal activity, employment and treatment history and the transition probabilities are dependent on age, gender, and race and treatment history.

#### Other models

There are other ad-hoc modelling approaches, which are designed and used by researchers to conduct economic evaluations in particular circumstances that do not fit in the mainstream modelling paradigms. Two such papers are briefly described. Rydell et al. [38] modelled demand in the cocaine market that depended on the number of users, initiation rate, escalating and de-escalating use rates; and supply in the cocaine market that depended on the production amount minus the seized amount. The model was used to estimate the impact of different interventions such as treatment or law enforcement on supply and demand, from which different cost-effectiveness indicators can be compared. Magnus et al. [39] estimated the economic benefits to health, productivity and leisure due to a reduction of alcohol consumption with reference to the Australian population in 2008. They collected data about health and employment corresponding to different levels of alcohol consumption, from which economic benefits at different levels of alcohol consumption were estimated. These models provide population based evaluation outcomes, but do not take into account the complexity of disease evaluation such as interaction, recurrent events and heterogeneity of study subjects.

### Perspectives on costs and benefits

The majority of the reviewed papers adopted a health care sector perspective (*n* = 26). The remaining papers adopted a broader perspective of costs and benefits by incorporating the social costs such as the impact of crime and benefits of employment in the evaluations of treatments [2, 3]. In assessing treatment interventions for alcohol and drug problems, it is clear that the selected perspective will impact on which costs and benefits are chosen and might impact on the assessment of net benefits of treatments enormously.

### Time frame

The use of modelling permits examination of a longer time frame for economic outcomes, superior to economic evaluations conducted on individual RCT’s with foreshortened timeframes. When the process is protracted as is frequently the case with dependence on drugs or alcohol, and thus where the costs and benefits of treatment are cumulative a longer timeframe is more appropriate. For the policy maker, this means economic projections into the distant future (and beyond the electoral cycle). The time frames used in the selected papers vary: there are six papers used 0-1 years; nine papers used 1-10 years and 23 papers used 10 years to life time. The majority of papers used cohort Markov, ISM and System Dynamics adopted long-term timeframe (e.g. ten years and up to lifetime). Zarkin et al. [2] illustrated that the choice of timeframe significantly influences the results in their study about the benefits and costs of methadone treatment in the U.S. They found that the benefit-cost ratio of methadone treatment produced from the lifetime model (37.72:1) was far more than the benefit-cost ratio from a static model of a one-off intervention (4.86:1).

## Discussion and conclusions

Economic evaluations that incorporate modelling play an important role in producing realistic evidence for policy making around health interventions for drug and alcohol problems. The complexity of including the resource implications across multiple domains, including behaviours such as initiating drug use, increasing use, cycling in and out of treatment or prison during which previous events may alter the drug users’ future life trajectories and affect the success of future treatments makes modelling in this field particularly challenging.

The decision tree modelling approach is a relatively easy technique for comparing different health interventions across a wide range of topics; however, it is not an optimal method when the population or treatment effects are heterogeneous, or when there are recurrent events, or when the outcome of interest is long-term. To this end, decision tree modelling may not be sufficient for evaluating treatments for alcohol and drugs.

On the other hand, the cohort Markov modelling maybe sufficiently sophisticated in that it may be able to replicate many of the potential developments in a disease process through the use of a series of health states. Particularly, it can account for both the time dimension by moving participants over time (e.g. time steps) and the heterogeneity of cohort by allocating them into relevant (but limited) states (e.g. poor health vs good health; types of treatment; drug dependent vs. not drug dependent). Attaching associated costs and benefits to states enables economic evaluations of various health interventions to be conducted. Although permitting more complex analyses than decision tree models, the Markov model relies on some major simplifications. First, it does not take into account the memory of past events that may shape participants’ future transitions. In other words, the transition probabilities from one state to other possible states are fixed at any time in the model regardless of individual histories. Thus, these models cannot account for the number or length of previous treatment episodes. Secondly, it is often not feasible to take into account the full spectrum of heterogeneity of the population of interest in a Markov model. Again, this is a limitation if it is expected that these factors will significantly impact on the outcomes or costs.

In the area of interventions for drug and alcohol problems, the individual based model is a powerful tool as it is possible to simulate multiple events such as initiating heroin use, becoming dependent, cycling in and out of various modalities of treatment, entering and exiting prison over a period of time. The shortcomings in the Markov model are addressed by (i) incorporating the past events and personal attributes in determining transition probabilities; (ii) simulating participants’ life trajectories individually rather than allocating participants into compartments; (iii) being flexible in time steps permitting participants to transit to the next state at different time intervals; (iv) providing a long-term perspective on costs and benefits of a series of health interventions over the participants’ life time. However, a challenge Individual-based model is that it requires a large number of parameters to characterise the complexity of transitions across states and at different times and dependence on individual attributes.

The selection of a modelling approach is likely subject to the costs and time of model building. When evidence is required quickly, a decision tree can provide an initial estimation. However, when the problem pertains to multiple domains such as scaling up OST treatment and with substantial budget allocation, more complex models such as ISM or DES would be preferable.

The choice of an appropriate modelling approach, time frame and perspective of costs and benefits for a study may change the modelling results and policy implications significantly. Despite there is ample literature about the guidelines to identify an appropriate modelling framework [20], there is not sufficient justification and discussion about the choice of modelling aspects to match the complexity of an intervention in the reviewed papers. It is not uncommon to see different modelling approaches, time frames and perspectives of costs and benefits were used to examine a similar type of research questions. For example, in regarding to economic evaluation of methadone treatment, the time frames ranged from 1 year [33] to life time [2]; various modelling approaches were used ranging from decision tree [32], cohort Markov [40] and individual-based microsimulation [2]; and both health care sector and societal perspectives of costs and benefits were used [2, 33]. The other study also concluded the same findings with this review, where the authors pointed out that there is no consistent measuring framework of costs and benefits regarding to alcohol treatment [41]. Therefore, there is a pressing need to improve the transparency in modelling practice by offering justification for the choice of modelling aspects. The characteristics and consequences of an intervention should be discussed and the choice of modelling aspects should be justified. The interested readers may consult literature about modelling guidelines in health economics to aid their decision [20–22].

A limitation of this review is that it only focused on three main aspects used in study that include modelling approach, time frame and perspectives of costs and benefits, but not the process of decision about the choices of modelling aspects. Although this review have critically appraised a number of papers with respect to the appropriateness of the above modelling aspects, future research should evaluate the characteristics, effects and consequences of the intervention and recommend suitable modelling approach, time frame and perspectives of costs and benefits. This will provide evidence about the difference between the ideal model and the chosen model in the research of drug and alcohol intervention.

## Additional file

Additional file 1: References to the list of selected papers for review. (DOC 84 kb)

## Abbreviations

ABM: agent-based models
ART: antiretroviral therapy
CEA: cost-effectiveness analysis
DES: discrete event simulations
MMT: methadone maintenance treatment
OST: opioid substitution treatment
QALYs: quality-adjusted life years
RCTs: randomised control trials
SD: system dynamics models
