# Supplementary material for: A systematic review of modelling approaches in economic evaluations of health interventions for drug and alcohol problems
Source: BMC Health Serv Res. 2016 Apr 13;16:127. doi: 10.1186/s12913-016-1368-8 (PMC4831174; doi:10.1186/s12913-016-1368-8)
Supplement: Additional file 1: — References to the list of selected papers for review. (DOC 84 kb) [file 12913_2016_1368_MOESM1_ESM.doc]

**Additional file 1: References to the list of selected papers for review**

Adi Y, Juarez-Garcia A, Wang D, et al. Oral naltrexone as a treatment for relapse prevention in formerly opioid-dependent drug users: a systematic review and economic evaluation. Health technology assessment (Winchester, England). 2007; 11: iii-iv, 1-85.

Alistar SS, Owens DK, Brandeau ML. Effectiveness and cost effectiveness of expanding harm reduction and antiretroviral therapy in a mixed HIV epidemic: a modeling analysis for Ukraine. PLoS Medicine / Public Library of Science. 2011; 8: e1000423.

Barbosa C, Taylor B, Godfrey C, et al. Modelling lifetime QALYs and health care costs from different drinking patterns over time: A Markov model. International Journal of Methods in Psychiatric Research. 2010; 19: 97-109.

Barnett PG, Zaric GS, Brandeau ML. The cost-effectiveness of buprenorphine maintenance therapy for opiate addiction in the United States. Addiction (Abingdon, England). 2001; 96: 1267-78.

Barnett PG. The cost-effectiveness of methadone maintenance as a health care intervention. Addiction (Abingdon, England). 1999; 94: 479-88.

Bayoumi AM, Zaric GS. The cost-effectiveness of Vancouver's supervised injection facility. CMAJ : Canadian Medical Association journal = journal de l'Association medicale canadienne. 2008; 179: 1143-51.

Cartwright, W. S. (2000). Cocaine medications, cocaine consumption and societal costs. *PharmacoEconomics, 18*(4), 405-413.

Coffin PO, Sullivan SD. Cost-Effectiveness of Distributing Naloxone to Heroin Users for Lay Overdose Reversal. Annals of Internal Medicine. 2013; 158: 1-U42.

Downs SM, Klein JD. Clinical preventive services efficacy and adolescents' risky behaviors. Archives of Pediatrics & Adolescent Medicine. 1995; 149: 374-9.

Kapoor A, Kraemer KL, Smith KJ, et al. Cost-effectiveness of screening for unhealthy alcohol use with % carbohydrate deficient transferrin: results from a literature-based decision analytic computer model. Alcoholism: Clinical & Experimental Research. 2009; 33: 1440-9.

Magnus A, Cadilhac D, Sheppard L, et al. The Economic Gains of Achieving Reduced Alcohol Consumption Targets for Australia. American Journal of Public Health. 2012; 102: 1313-19.

Mortimer D, Segal L. Economic evaluation of interventions for problem drinking and alcohol dependence: Cost per QALY estimates. Alcohol and Alcoholism. 2005; 40: 549-55.

Navarro HJ, Shakeshaft A, Doran CM, et al. The potential cost-effectiveness of general practitioner delivered brief intervention for alcohol misuse: Evidence from rural Australia. Addictive behaviors. 2011; 36: 1191-98.

Nosyk B, Guh DP, Bansback NJ, et al. Cost-effectiveness of diacetylmorphine versus methadone for chronic opioid dependence refractory to treatment. CMAJ Canadian Medical Association Journal. 2012; 184: E317-28.

Palmer AJ, Neeser K, Weiss C, et al. The long-term cost-effectiveness of improving alcohol abstinence with adjuvant acamprosate. Alcohol and Alcoholism. 2000; 35: 478-92.

Purshouse RC, Brennan A, Rafia R, et al. Modelling the cost-effectiveness of alcohol screening and brief interventions in primary care in England. Alcohol and Alcoholism. 2013; 48: 180-88.

Rydell, C.P, & Everingham, S.S. Controlling cocaine: Supply versus demand programs, Drug Policy Research Centre, RAND Corp. 1994.

Schackman BR, Metsch LR, Colfax GN, et al. The cost-effectiveness of rapid HIV testing in substance abuse treatment: Results of a randomized trial. Drug and alcohol dependence. 2013; 128: 90-97.

Sheerin IG, Green FT, Sellman JD. What is the cost-effectiveness of hepatitis C treatment for injecting drug users on methadone maintenance in New Zealand? Drug and alcohol review. 2004; 23: 261-72.

Tariq L, van den Berg M, Hoogenveen RT, et al. Cost-effectiveness of an opportunistic screening programme and brief intervention for excessive alcohol use in primary care. PLoS ONE [Electronic Resource]. 2009; 4: e5696.

Tran BX, Ohinmaa A, Duong AT, et al. Cost-effectiveness of integrating methadone maintenance and antiretroviral treatment for HIV-positive drug users in Vietnam's injection-driven HIV epidemics. Drug and alcohol dependence. 2012; 125: 260-6.

Tran BX, Ohinmaa A, Duong AT, et al. The cost-effectiveness and budget impact of Vietnam's methadone maintenance treatment programme in HIV prevention and treatment among injection drug users. Global public health. 2012; 7: 1080-94.

van den Berg M, van Baal PHM, Tariq L, et al. The cost-effectiveness of increasing alcohol taxes: a modelling study. Bmc Medicine. 2008; 6.

Vickerman P, Martin N, Turner K, et al. Can needle and syringe programmes and opiate substitution therapy achieve substantial reductions in hepatitis C virus prevalence? Model projections for different epidemic settings. Addiction (Abingdon, England). 2012; 107: 1984-95.

Wammes JJ, Siregar AY, Hidayat T, et al. Cost-effectiveness of methadone maintenance therapy as HIV prevention in an Indonesian high-prevalence setting: A mathematical modeling study. International Journal of Drug Policy. 2012; 23: 358-64.

Zaric GS, Brandeau ML. Optimal investment in a portfolio of HIV prevention programs. Medical Decision Making. 2001; 21: 391-408.

Zaric GS, Barnett PG, Brandeau ML. HIV transmission and the cost-effectiveness of methadone maintenance. American Journal of Public Health. 2000; 90: 1100-11.

Zaric GS, Brandeau ML, Barnett PG. Methadone maintenance and HIV prevention: A cost-effectiveness analysis. Management Science. 2000; 46: 1013-31.

Zarkin GA, Dunlap LJ, Hicks KA, et al. Benefits and costs of methadone treatment: results from a lifetime simulation model. Health economics. 2005; 14: 1133-50.

Zarkin G.A, Cowell A.J, Hicks K.A, et al. Lifetime benefits and costs of diverting substance-abusing offenders from state prison. *Crime & Delinquency. 2012;* XX(X): 1–22

Alistar SS, Owens DK, Brandeau ML. Effectiveness and cost effectiveness of oral pre-exposure prophylaxis in a portfolio of prevention programs for injection drug users in mixed HIV epidemics. PloS one. 2014; 9: e86584.

Angus C, Scafato E, Ghirini S, et al. Cost-effectiveness of a programme of screening and brief interventions for alcohol in primary care in Italy. BMC family practice. 2014; 15: 26.

Braithwaite RS, Nucifora KA, Kessler J, et al. How inexpensive does an alcohol intervention in Kenya need to be in order to deliver favorable value by reducing HIV-related morbidity and mortality? Journal of acquired immune deficiency syndromes (1999). 2014; 66: e54-8.

Ciketic S, Hayatbakhsh R, McKetin R, et al. Cost-effectiveness of counselling as a treatment option for methamphetamine dependence. Journal of Substance Use. 2015; 20: 239-46.

Jackson H, Mandell K, Johnson K, et al. Cost-Effectiveness of Injectable Extended-Release Naltrexone Compared With Methadone Maintenance and Buprenorphine Maintenance Treatment for Opioid Dependence. Substance abuse. 2015; 36: 226-31.

Laramee P, Brodtkorb TH, Rahhali N, et al. The cost-effectiveness and public health benefit of nalmefene added to psychosocial support for the reduction of alcohol consumption in alcohol-dependent patients with high/very high drinking risk levels: a Markov model. BMJ open. 2014; 4: e005376.

Schackman BR, Leff JA, Barter DM, et al. Cost-effectiveness of rapid hepatitis C virus (HCV) testing and simultaneous rapid HCV and HIV testing in substance abuse treatment programs. Addiction (Abingdon, England). 2015; 110: 129-43.

Thanh NX, Jonsson E, Moffatt J, et al. An economic evaluation of the parent-child assistance program for preventing fetal alcohol spectrum disorder in Alberta, Canada. Administration and policy in mental health. 2015; 42: 10-8
